# Association of dietary total antioxidant capacity with depression, anxiety, and sleep disorders: A systematic review of observational studies

**Published:** 2021-09-27

**Authors:** Gabriela Amorim Pereira, Alessandra da Silva, Helen Hermana M. Hermsdorff, Ana Paula Boroni Moreira, Aline Silva de Aguiar

**Affiliations:** ^1^Department of Collective Health, Faculty of Medicine, Universidade Federal de Juiz de Fora, Juiz de Fora, Minas Gerais, Brazil; ^2^Department of Nutrition and Health, Laboratory of Energy Metabolism and Body Composition, Universidade Federal de Viçosa, Viçosa, Minas Gerais, Brazil; ^3^Department of Nutrition, Universidade Federal de Juiz de Fora, Juiz de Fora, Minas Gerais, Brazil

**Keywords:** antioxidants, common mental disorders, dietary total antioxidant capacity, mental health, nutrition

## Abstract

**Background and Aim:**

We aimed to systematically review observational studies that evaluated the potential association of the dietary total antioxidant capacity (dTAC) with common mental disorders (depression and anxiety) and sleep disorders.

**Methods:**

Studies with an observational design that evaluated the association between the dTAC and common mental disorders and sleep disorders were identified using the PubMed and Scopus databases. The meta-analysis guideline of observational studies in epidemiology and the preferred reporting items for systematic reviews and meta-analysis were used to conduct and report the data of this systematic review.

**Results:**

Of the 439 records, seven studies were included in this review. There was a sample variation of 41-3297 participants. We highlight that five of the studies analyzed were conducted in the Iranian population. Four studies analyzed only women, and three studies were conducted with postmenopausal or climacteric women. Four cross-sectional studies showed inverse associations between the dTAC and depression, anxiety, and sleep disorders in Iranians.

**Conclusion:**

The consumption of a diet rich in antioxidants, characterized by high dTAC scores, seems to be inversely associated with depression, anxiety, and sleep disorders. However, further studies with different populations and designs are necessary for a better understand this relationship.

**Relevance to Patients:**

This review assesses the association of the dTAC with common mental disorders (depression and anxiety) with sleep disorders. This will help guide further studies on the relationship between diet and mental disorders and sleep disorders. Knowledge about these relationships is essential for the creation of non-pharmacological practices for the prevention of these disorders.

## 1. Introduction

Depression and anxiety are common mental disorders due to their high prevalence in the contemporary society [1]. Depression has been diagnosed in more than 322 million individuals worldwide and is one of the major contributors to the global burden of disease [1,2]. Anxiety disorders have affected approximately 264 million people worldwide. Although anxiety is an important and necessary feeling in certain situations, it may be indicative of mental disorders when observed to be in an uncontrolled degree [1]. This condition can be classified into generalized anxiety disorders, panic syndrome, and obsessive-compulsive disorder among others [1,3].

Sleep disorders can be characterized as manifestations that cause impairment in the sleep quality, among which changes in the circadian rhythm and insomnia stand out [4,5]. Such disorders are risk factors for the development of anxiety and depression; In addition, such sleep disorders may arise as a consequence of common mental disorders [4,5]. Notably, insomnia affects approximately 6 to 10% of the general population. Nowadays, both sleep disorders and depressive and anxiety disorders have been evidenced as indirect effects of the COVID-19 pandemic [6-8].

The occurrence of common mental disorders and sleep disorders may have multiple etiologies, including behavioral, biological, social, and psychological factors [1,9,10]. Oxidative stress is generated by an imbalance between the reactive oxygen species (ROS) and enzymatic and non-enzymatic antioxidant defense systems [11,12]. Since oxidative stress can impair the neuronal and neurotransmitter function and lead to a dysregulation of circadian rhythms, it has also been linked to the pathophysiology of mental disorders and sleep disorders [11-14]. Thus, oxidative stress reduction may mediate protection against common mental disorders [15].

Antioxidant dietary factors are the main external contributors to the non-enzymatic antioxidant defense [16]. Studies have shown associations between antioxidant compounds, such as Vitamin C, Vitamin A, polyphenols, beta-carotene, and mental health [17-19]. Moreover, the interaction between the different antioxidants in the diet appears to be more effective than the action of an isolated nutrient [20-22].

The dietary total antioxidant capacity (dTAC) is the sum of all or most antioxidants consumed, which estimates the cumulative effect of the antioxidants in the overall diet [23]. Studies have shown an inverse relationship between higher dTAC values and the risk of developing chronic diseases [24], central obesity, and oxidative stress markers [20]. The relationship between the dTAC and depression and anxiety scores [25-30] and sleep disorders [26,31] have currently been investigated; however, the results are controversial. Thus, we systematically reviewed observational studies that evaluated the potential association between the dTAC and common mental disorders (depression and anxiety) and sleep disorders.

## 2. Materials and Methods

### 2.1. Protocol and registration

To conduct and report the data of this systematic review, the meta-analysis guidelines of observational studies in epidemiology [32] and preferred reporting items for systematic reviews and meta-analysis were used [33]. This review was registered in PROSPERO (www.crd.york.ac.uk/prospero/) under the number CRD42020212014.

### 2.2. Eligibility criteria

In this review, the inclusion criteria were original observational articles in humans that related the dTAC with depression, depressive symptoms, anxiety, or sleep disorders. In addition, studies that reported the associations of data as b-values or odds ratios followed by the 95% confidence interval were included, as well as studies that reported the data as measures of central tendency and dispersion. The studies that reported dTAC data in averages, tertiles, quartiles, and quintiles were also included. The criteria for non-inclusion in the systematic review were review articles, letters, book chapters, articles with animals, articles that did not analyze the dTAC, and the outcomes of interest. The review did not include language or date restrictions.

### 2.3. Search strategies

Two researchers (GAP and AS) independently conducted the search for original observational studies that evaluated the association between dTAC and depression or depressive symptoms, anxiety or anxiety related symptoms, and sleep disorders. To identify articles according to the inclusion criteria, we searched the online databases PubMed/Medline (https://pubmed.ncbi.nlm.nih.gov/advanced/) and Scopus (https://www.scopus.com/home.uri) between October 2020 and July 2021. An exhaustive literature review was conducted with the following search terms: “Total dietary antioxidant capacity;” “Dietary total antioxidant capacity;” and “Non enzymatic antioxidant capacity.” The search terms were selected from previous readings of published manuscript that related dTAC and health outcomes.

### 2.4. Selection of studies and data extraction

The selection of studies was based on the analysis of titles, abstracts, and full texts by two independent authors (GAP and AS). Duplicate articles were manually identified. Consensus between the authors resolved divergent decisions. In the absence of the full article or sufficient information to interpret the articles, we contacted the corresponding author to request such information. From the eligible studies, the two authors (GAP and AS) independently extracted the following information: (i) Name of the first author, year, and country where the study was conducted; (ii) sample characteristics; (iii) methodology to quantify dTAC and to evaluate the outcomes; (iv) adjustment variables used in the analyses; and (v) main results.

### 2.5. Evaluation of the quality of studies

The National Heart, Lung, and Blood Institute study quality assessment tools were used [34]. The tool for evaluating cohort and cross-sectional studies was composed of 14 criteria, and questions 6, 7, 10, and 13 were applicable only to the cohort studies. In turn, the tool for assessing the quality of case-control studies was composed of 12 criteria. In both tools, for each question, the score 1 was considered in case of a positive answer “yes.” A zero score has been assigned for “no” answers, which can be “not applicable,” “not reported,” or “not possible to determine” questions. These evaluations were not a decision criterion for the inclusion of the studies, but rather to contribute to the evaluation of the quality of the evidence pointed out by each one.

We classified the studies as good, fair, or poor according to the evaluation of the quality of the studies applied by two authors (GAP and AS). Consensus resolved divergences regarding the studies score and quality classification. The main criteria considered for classifying the studies were the use of validated measures for results and exposure, a clearly defined study population, the use of possible confounding variables, and the evaluation of the participation rate in the study.

## 3. Results

### 3.1. Search

**Figure 1:**
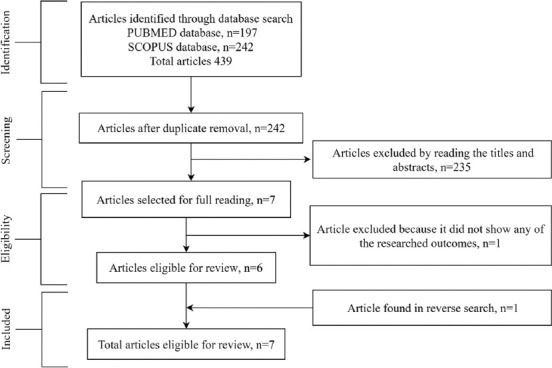
Stages of identification, inclusion, and exclusion of articles in the review

Of the 439 articles from the two databases, 197 articles were duplicates. Of the remaining 242 articles, 235 articles were excluded after reading the titles and abstracts. Subsequently, a total of seven articles were selected for full reading. After reading, one article was excluded because it did not present any outcome of interest (depression, anxiety, or sleep disorders). From the list of references of the six remaining articles, a reverse search was performed, and one additional article was included. Thus, seven articles were selected for this review (Figure 1).

### 3.2. Characteristics of included studies

Of the seven studies included in this systematic review, five studies presented a cross-sectional design [25,26,28,29,31], one study was a case-control study [30], and one study was a prospective cohort study with 3 years follow-up [27]. The sample size ranged from 41 [29] to 3,297 participants [28]. Regarding the studied population, four studies investigated associations only in women, three studies in postmenopausal or climacteric women [25,29,31], and one in adult or elderly women with Type 2 diabetes [26]. Two studies analyzed both men and women, one with young, adult, and elderly workers [27], and the other with apparently healthy adults [28]. Finally, one study analyzed only young men [30].

Regarding the origin of the studies, five studies were conducted with Iranian participants [25,26,28,30,31], one study with Brazilian participants [29], and one study with Japanese participants [27].

To determine the dTAC, two studies used the oxygen radical absorbance capacity (ORAC) and ferric reducing antioxidant power (FRAP) assays [26,27]; one study used the Trolox equivalent antioxidant capacity (TEAC) and FRAP assays [30]; two studies used only ORAC [25,31]; one study used the vitamin C equivalent antioxidant capacity (VCAC) method [29]; and one study analyzed only the FRAP [28].

Regarding the tools used to measure the outcomes, Daneshzad *et al*. (2020) analyzed the sleep quality using the Pittsburgh Sleep Quality Index and symptoms of depression and anxiety using the depression, anxiety, and stress scale (DASS-21) [26]. Abshirini *et al*. (2018) observed the presence of depressive mood, anxiety symptoms, and sleep problems using the menopause rating scale [31]. Abshirini *et al*. (2019) used the DASS-42 [25]. The Hospital Anxiety and Depression Scale questionnaire [28], Center for Epidemiological Studies Depression Scale [27], and Beck Depression Inventory-II (BDI-II) [30] were also used. In addition, Oliveira *et al*. (2019) used the self-report of medical diagnosis of depression and for measuring the depressive symptoms used BDI [29].

### 3.3. Results of individual studies

Of the seven studies included in this systematic review, four studies observed an inverse association between the dTAC and the outcomes of interest (Table 1) [25,26,28,31].

**Table 1:** Characteristics of the seven studies included in the systematic review

Authors/Year of publication/Country	Sample characteristics	Objective of the study	Methodology	Adjustment variables	Main results
Abshirini *et al*., 2018 Iran	n: 400 postmenopausal women (adults and older adults)	To assess the association between dTAC and menopausal symptoms in postmenopausal middle-aged women	Assay: ORAC Assessment of food consumption: validated semi-quantitative food frequency questionnaire with 147 food items Outcome assessment: Menopause rating scale questionnaire	Age, educational level, waist circumference, physical activity, use of dietary supplements, fiber, tea, coffee, and energy intake	•Higher ORAC quartile was associated with lower chance of anxiety symptoms, sleep problems, irritability, exhaustion/difficulty concentrating Absence of association between ORAC and depressed mood
Abshirini *et al*., 2019 Iran	n: 175 postmenopausal women (adults and older adults)	To evaluate the association between dTAC with scores of depression, stress, anxiety, and oxidative stress in postmenopausal women	Assay: ORAC Assessment of food consumption: validated semi-quantitative food frequency questionnaire with 147 food items Outcome assessment: Stress, Anxiety and Depression Scale (DASS-42)	Age, time of menopause; education level, waist circumference, physical activity, use of dietary supplements, fiber, energy, and coffee intake	• Inverse association between ORAC and depression and anxiety scores • Absence of association between dTAC and stress score
Daneshzad *et al*., 2020 Iran	n: 265 type 2 diabetic women (adults and older adults)	To evaluate the association between dTAC with sleep, stress, anxiety, and depression in women	Assay: ORAC and FRAP Assessment of food consumption: semi-quantitative food frequency questionnaire validated with 168 food items. Outcome assessment: Pittsburgh Sleep Quality Index and Depression, Anxiety and Stress Scale (DASS-21)	Age, BMI, energy intake, physical activity, blood pressure, medication, supplement consumption, socioeconomic classification, nap times, hours of night sleep	•Higher tertile of FRAP and ORAC was associated with lower chances of sleeping poorly, of depressive symptoms and stress Higher FRAP tertile was associated with lower chance of anxiety
Milajerdi *et al*., 2018 Iran	n: 3.297 men and women, apparently healthy adults	Investigate the association between dTAC and depression and anxiety among Iranian adults	Essay: FRAP Assessment of food consumption: validated semi-quantitative food frequency questionnaire with 106 food items Outcome assessment: Hospital Anxiety and Depression Scale	Age, sex, energy consumption, marital status, socioeconomic status, smoking, presence of chronic conditions, physical activity, use of supplements, antidepressant medication use, intake of omega-3 fatty acids and BMI	Higher FRAP quintile was associated with lower chance of higher depression and anxiety scores
Miki *et al*., 2020 Japan	n: 911 men and women (youth, adults, and older adults)	To assess the association of dTAC and the incidence of depressive symptoms in Japanese workers	Assay: ORAC and FRAP Assessment of food consumption: Brief, validated dietary questionnaire with 58 food items Outcome assessment: Japanese version of the scale of the left for Epidemiological Studies of depression Duration: 3 years of follow-up	Age, sex, marital status, degree of employment, night work or on a rotating shift, overtime work, Job strain, physical activity at work, household chores, commuting, or leisure, smoking; BMI, consumption of alcohol, total energy intake, antioxidant supplement use, intake of folate, vitamin B6, vitamin B12, n-3 polyunsaturated fatty acids, magnesium, and zinc, and CES-D score	Absence of association between ORAC and FRAP with incidence of depressive symptoms
Oliveira *et al*., 2019* Brazil	n: 41 climacteric women	To evaluate the possible relationship between dTAC and polyphenol intake and depression in climacteric women	Assay: VCAC Assessment of food consumption: 24 h dietary recall Outcome assessment: Medical diagnosis of depression and Beck Depression Inventory	No adjustment	Absence of difference in average VCAC values in women with depression or no
Prohan *et al*., 2014* Iran	n: 60 men (young) cases: 30 students diagnosed with depression control: 30 healthy students	To evaluate associations between dTAC and serum CAT with depression scales in young university students	Essay: TEAC and FRAP Assessment of food consumption: Two 24 h dietary recalls and validated semi-quantitative food frequency questionnaire with 168 food items Outcome assessment: Beck-II Depression Inventory	No adjustment	Absence of difference in the average values of TEAC and FRAP between cases and controls

* Did not assess association through regression analyzes, dTAC: Dietary total antioxidant capacity; n: Sample size; BMI: Body mass index; ORAC: Oxygen radical absorbance capacity; FRAP: Ferric reducing ability of plasma; VCAC: Vitamin C equivalent antioxidant capacity; TEAC: Trolox equivalent antioxidant capacity; CES-D: center for epidemiologic studies depression scale

#### 3.3.1. Cross-sectional studies

Type 2 diabetic women, who were classified in the highest tertiles of FRAP and ORAC exhibited a 94% and 87% lower chance of sleeping poorly, respectively. They also had lower chances of depression compared to the first tertile. The highest FRAP tertile was associated with a lower chance of anxiety than the first tertile. These results were independent of age, BMI, energy consumption, physical activity, blood pressure, medications, supplement consumption, socioeconomic classification, nap times, and hours of night sleep [26].

Two studies analyzed post-menopausal women [25,31]. In the first study, they observed no significant association between the dTAC and depressed mood. However, women in the last quartile of ORAC had a 71% lower chance of sleep problems and 62% less chance of anxiety. These findings were independent of age, education, waist circumference, physical activity, use of supplements, fiber consumption, tea and coffee consumption, and total energy intake [31]. In the second study, an inverse association was observed between the ORAC and depression and anxiety scores [25].

Milajerdi *et al*. (2019) observed that Iranian adults included in the highest quintile of FRAP presented 43% and 38% lower chances of depressive and anxiety symptoms, respectively, when compared to the first FRAP quintile. These associations were independent of age, sex, energy intake, omega-3 fatty acid consumption, marital status, socioeconomic status, smoking, presence of chronic conditions, physical activity, the use of supplements, antidepressant drugs, and BMI. In contrast, a Brazilian study found no difference in the mean dTAC values between climacteric women with and without depression [29].

#### 3.3.2. Prospective cohort study

A prospective cohort study investigated the incidence of depressive symptoms in Japanese workers of both sexes, over a period of 3 years. They noted no association between the FRAP and ORAC and depressive symptoms in crude and multivariate analyses [27].

#### 3.3.3. Case-control study

A case-control study investigated young men with high scores for depressive symptoms and men free of depressive symptoms as the control. They observed no significant differences in the FRAP and TEAC means between the cases and controls. There was no association between the dTAC and the depressive symptom scores [30].

### 3.4. Quality of studies

Among the analysis of cross-sectional studies, three studies scored seven points. They were classified as fair because they did not report the participation rate in the study and did not justify the sample size [25,26,31]. A cross-sectional study received eight points. This study was considered as good; however, there was no justification for the sample size [28]. In turn, a cross-sectional study with six points was classified as poor. Its outcome was self-reported and not validated; in addition, the confounding factors were not considered [29]. A cohort study obtained 10 points (good); however, there was a loss of follow-up over 20% of the initial population [27]. The case-control study scored seven (poor) because possible confounding variables were not considered in the dTAC comparison between the cases and controls (Tables 2 and 3) [30].

**Table 2:** Quality assessment of cross-sectional and cohort studies

Study	Questions														Overall rating

	1	2	3	4	5	6	7	8	9	10	11	12	13	14	
Daneshzad *et al*., 2020	Y	Y	N	Y	N	N	N	Y	Y	N	Y	N	N	Y	Fair
Abshirini *et al*., 2018	Y	Y	N	Y	N	N	N	Y	Y	N	Y	N	N	Y	Fair
Abshirini *et al*., 2019	Y	Y	N	Y	N	N	N	Y	Y	N	Y	N	N	Y	Fair
Oliveira *et al*., 2019	Y	Y	N	Y	N	N	N	Y	Y	N	N	N	N	N	Poor
Milajerdi *et al*., 2018	Y	Y	Y	Y	N	N	N	Y	Y	N	Y	N	N	Y	Good
Miki *et al*., 2020	Y	Y	Y	Y	N	Y	Y	Y	Y	N	Y	N	N	Y	Good
Legends: Y: Yes; N: No: Not applicable, not reported, not possible to determine.															
Questions															
1. Was the research question or objective in this paper clearly stated?															
2. Was the study population clearly specified and defined?															
3. Was the participation rate of eligible persons at least 50%?															
4. Were all the subjects selected or recruited from the same or similar populations (including the same time period)? Were inclusion and exclusion criteria for being in the study prespecified and applied uniformly to all participants?															
5. Was a sample size justification, power description, or variance and effect estimates provided?															
6. For the analyses in this paper, were the exposure (s) of interest measured prior to the outcome (s) being measured?															
7. Was the timeframe sufficient so that one could reasonably expect to see an association between exposure and outcome if it existed?															
8. For exposures that can vary in amount or level, did the study examine different levels of the exposure as related to the outcome (e.g., categories of exposure, or exposure measured as continuous variable)?															
9. Were the exposure measures (independent variables) clearly defined, valid, reliable, and implemented consistently across all study participants?															
10. Was the exposure (s) assessed more than once over time?															
11. Were the outcome measures (dependent variables) clearly defined, valid, reliable, and implemented consistently across all study participants?															
12. Were the outcome assessors blinded to the exposure status of participants?															
13. Was loss to follow-up after baseline 20% or less?															
14. Were key potential confounding variables measured and adjusted statistically for their impact on the relationship between exposure (s) and outcome (s)?															

**Table 3:** Quality assessment of cross-sectional and cohort studies

Study	Questions												Overall rating

	1	2	3	4	5	6	7	8	9	10	11	12	
Prohan *et al*., 2014	Y	Y	N	Y	Y	Y	N	Y	N	Y	N	N	Poor
Legends: Y: Yes; N: No: Not applicable, not reported, not possible to determine.													
Questions													
1. Was the research question or objective in this paper clearly stated and appropriate?													
2. Was the study population clearly specified and defined?													
3. Did the authors include a sample size justification?													
4. Were controls selected or recruited from the same or similar population that gave rise to the cases (including the same timeframe)?													
5. Were the definitions, inclusion and exclusion criteria, algorithms or processes used to identify or select cases and controls valid, reliable, and implemented consistently across all study participants?													
6. Were the cases clearly defined and differentiated from controls?													
7. If less than 100 percent of eligible cases and/or controls were selected for the study, were the cases and/or controls randomly selected from those eligible?													
8. Was there use of concurrent controls?													
9. Were the investigators able to confirm that the exposure/risk occurred prior to the development of the condition or event that defined a participant as a case?													
10. Were the measures of exposure/risk clearly defined, valid, reliable, and implemented consistently (including the same time period) across all study participants?													
11. Were the assessors of exposure/risk blinded to the case or control status of participants?													
12. Were key potential confounding variables measured and adjusted statistically in the analyses? If matching was used, did the investigators account for matching during study analysis?													

## 4. Discussion

It has been suggested that the ingestion of dietary antioxidants may protect against oxidative damage and related clinical complications [25,26,28,31]. To our knowledge, this study is the first to review the relationship between the dTAC and common mental disorders (depression and anxiety) and sleep disorders. We observed that four cross-sectional studies reported an inverse association between the dTAC and depression or anxiety scores, or sleep disorders in Iranians [25,26,28,31].

**Figure 2:**
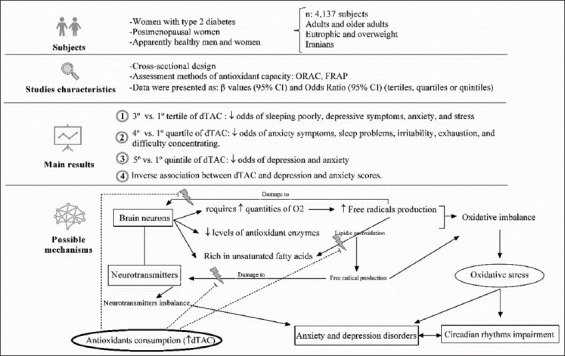
Figure summary of the four studies that observed a significant relationship between the total antioxidant capacity of the diet (dietary total antioxidant capacity) and the outcomes evaluated in this systematic review. Legend: ↓ = low, ↑ high, dashed lines = cancel the effect.

In fact, the brain is highly vulnerable to oxidative damage due to the characteristics of the organ itself, such as a high cellular metabolic rate and a constitution rich in lipids and unsaturated fatty acids, which are substrates for oxidation [12,13,35,36]. Brain neurons have a high metabolic demand and lower endogenous levels of antioxidants compared to other cells with an equivalent metabolism [37]. It is noteworthy that the redox imbalance in the brain may be related to risk factors for depression and anxiety, such as increased inflammation, impaired neuronal plasticity, and reduced neuronal signaling [13,36]. It is important to show that oxidative and inflammatory processes are interconnected in the pathogenesis of depression. During the inflammatory process, increases in interleukins are able to activate the activity of the enzyme indoleamine 2,3-dioxygenase (IDO), which is involved in the synthesis of kynurenine (KYN) from tryptophan, diverting it from its pathway. In addition to reducing the serotonin synthesis, the activation of the KYN pathway generates catabolites called TRYCATS, which induce the influx of calcium, which in turn, generates mitochondrial dysfunction and compromises the cellular antioxidant system (Figure 2) [36].

It is highlighted that sleep deprivation can act as a factor that increases the oxidative stress. Thus, in alertness, there is a high neuronal metabolism, a greater requirement for oxygenation, and consequently, a greater formation of ROS. During sleep, there is an increase in the antioxidant state that promotes brain protection [38]. However, it is possible that oxidative stress affects the cellular ability to regulate circadian rhythms [14].

Notably, the diet contributes directly to the composition of the non-enzymatic antioxidant defense and maintenance of the redox balance, in addition to antioxidant enzymes [16]. The consumption of antioxidants, such as carotenoids, flavonoids, and Vitamins E and C, can reduce the ROS and consequently, prevent oxidative damage [26]. Thus, a higher dTAC is related to a higher consumption of these antioxidants, which can exert beneficial effects on mental health [26,28]. In this sense, therapies containing antioxidants, such as vitamins C and E, can help in the treatment of psychiatric disorders [13]. Furthermore, a diet rich in antioxidants, such as polyphenols, could be a good strategy for preventing anxiety and depression (Figure 2) [18,39].

Furthermore, the anti-inflammatory effect of a high dTAC may explain its inverse association with mental disorders, in addition to antioxidant protection [25,40]. In this sense, studies have shown that a higher dTAC is associated with a lower dietary inflammatory index and decreased systemic inflammation markers [41-43]. In addition, individuals with a higher dTAC may have a diet with a greater consumption of fruits, vegetables, fibers, and vitamins [20,26,28]. These food groups offer a better quality of the diet, which can offer a potential protective effect against common mental disorders [26,44,45]. In addition to its anti-inflammatory effects, the consumption of certain foods may be related to increased melatonin concentrations. In this sense, a review by Pereira *et al*. (2020) showed that the consumption of melatonin source foods, such as cherries, grapes, bananas, pineapples, and dark green vegetables was related to the increased urinary excretion of the melatonin metabolite, 6-sulfatoxymelatonin, or circulating melatonin [46]. This indolamine is a potent antioxidant that coordinates the synchronization of circadian rhythms and controls the onset and quality of sleep [46,47]. Thus, the consumption of food sources of this indoleamine has been related to improvements in the sleep quality and increased urinary antioxidant capacity [48-50].

Of the five studies with Iranian subjects, four studies reported an inverse association between the dTAC and depression and anxiety scores or sleep disorders. Of these, three studies were classified as fair [25,26,31], and only one study was classified as good [28]. Notably, the study that found no association was classified as poor [51]. On analyzing the nationality of the studies that found significant associations, it is important to highlight that the cultural and environmental factors, such as planting, cooking methods, and typical culinary, can influence the antioxidant content of the diet [52]. In addition, three studies analyzed only women [25,26,31]. There is a greater prevalence of depression, anxiety, and sleep disorders among women, which is associated with increasing age and menopause. Menopausal women may experience hormonal, mood, body metabolism, and lifestyle changes [53,54]. Moreover, a case-control study in which no associations were found was conducted with young Iranian men. Confounding variables were not used to investigate the differences in the mean dTAC between the cases and controls [30]. Thus, further investigation of the relationship between the dTAC and mental and sleep disorders according to the sex, age, and ethnicity is necessary. Another point to be considered is the possible reverse causality in cross-sectional studies, since hormonal changes from sleep and mental disorders can lead to worse food choices [55,56].

Unlike the studies on Iranian women, a study with climacteric Brazilians found no association between the dTAC and depression [29]. In this Brazilian study, a self-reported diagnosis of depression was not validated, and adjustments for possible confounding variables were not used. In addition, only a 24-h food recall was used for the dietary assessment [29]. Thus, it was not possible to accurately measure the dTAC from the diet because the variability in antioxidant food intake on different days was not considered [57].

In turn, no association was found in a prospective cohort of Japanese workers classified as of good quality [27]. Among the studies analyzed here, the one that offered the best level of evidence and some notable points should be highlighted. In this study, the authors made adjustments to the consumption of vitamins and minerals (folate, Vitamin B6, Vitamin B12, magnesium, and zinc) because they could act as protective factors for depressive symptoms [27]. Another notable point in this study was that the associations between the dTAC and depression by dietary sources (foods and beverages) were investigated. Therefore, there may be a greater bioavailability of antioxidants according to the food sources [27,58].

### 4.1. Limitations of the current research and challenges ahead

Although this systematic review supports an inverse association between the dTAC with common mental disorders and sleep disorders, we observed a limited number of available studies on this topic. A low sample size was observed in most studies and the majority of the studies were conducted with Iranian individuals and with women, which can generate a bias when extrapolating the results. We also highlight that some of the studies presented methodological biases, which limit interpretations. Therefore, we reinforce the need to conduct studies with a large sample size, with different nationalities, and with designs that allow the inferring of causality for the relationship of dTAC with common mental disorders and sleep disorders.

## 5. Conclusion

Most of the reviewed studies reported an inverse association between the dTAC with common mental disorders and sleep disorders. We encourage further research to better understand this relationship and the mechanisms involved, with different assessment methods, additional study countries and with larger sample sizes.

## References

[1] World Health Organization (2017). Depression and Other Common Mental Disorders.

[2] World Health Organization (2020). Depression.

[3] Spoorthy MS, Chakrabarti S, Grover S (2019). Comorbidity of Bipolar and Anxiety Disorders:An Overview of Trends in Research. World J Psychiatry.

[4] Okun ML, Mancuso RA, Hobel CJ, Schetter CD, Coussons-Read M (2018). Poor Sleep Quality Increases Symptoms of Depression and Anxiety in Postpartum Women. J Behav Med.

[5] Thorpy MJ (2012). Classification of Sleep Disorders. Neurotherapeutics.

[6] Perlis ML, Vargas I, Ellis JG, Grandner MA, Morales KH, Gencarelli A (2020). The Natural History of Insomnia:The Incidence of Acute Insomnia and Subsequent Progression to Chronic Insomnia or Recovery in Good Sleeper Subjects. Sleep.

[7] Han RH, Schmidt MN, Waits WM, Bell AK, Miller TL (2020). Planning for Mental Health Needs during COVID-19. Curr Psychiatry Rep.

[8] Vindegaard N, Benros ME (2020). COVID-19 Pandemic and Mental Health Consequences:Systematic Review of the Current Evidence. Brain Behav Immun.

[9] Humer E, Pieh C, Brandmayr G (2020). Metabolomics in Sleep, Insomnia and Sleep Apnea. Int J Mol Sci.

[10] LeBlanc M, Mérette C, Savard J, Ivers H, Baillargeon L, Morin CM (2009). Incidence and Risk Factors of Insomnia in a Population-Based Sample. Sleep.

[11] Bajpai A (2014). Oxidative Stress and Major Depression. J Clin Diagn Res.

[12] Bhatt S, Nagappa AN, Patil CR (2020). Role of Oxidative Stress in Depression. Drug Discov Today.

[13] Ng F, Berk M, Dean O, Bush AI (2008). Oxidative Stress in Psychiatric Disorders:Evidence Base and Therapeutic Implications. Int J Neuropsychopharmacol.

[14] Wilking M, Ndiaye M, Mukhtar H, Ahmad N (2013). Circadian Rhythm Connections to Oxidative Stress:Implications for Human Health. Antioxid Redox Signal.

[15] Jimenez-Fernandez S, Gurpegui M, Diaz-Atienza F, Perez-Costillas L, Gerstenberg M, Correll CU (2015). Oxidative Stress and Antioxidant Parameters in Patients with Major Depressive Disorder Compared to Healthy Controls before and after Antidepressant Treatment:Results from a Meta-Analysis. J Clin Psychiatry.

[16] Kruk J, Aboul-Enein HY, Kładna A, Bowser JE (2019). Oxidative Stress in Biological Systems and its Relation with Pathophysiological Functions:The Effect of Physical Activity on Cellular Redox Homeostasis. Free Radic Res.

[17] Park JY, You JS, Chang KJ (2010). Dietary Taurine Intake, Nutrients Intake, Dietary Habits and Life Stress by Depression in Korean Female College Students:A Case-Control Study. J. Biomed Sci.

[18] Gomez-Pinilla F, Nguyen TT (2012). Natural Mood Foods:The Actions of Polyphenols Against Psychiatric and Cognitive Disorders. Nutr Neurosci.

[19] Payne ME, Steck SE, George RR, Steffens DC (2012). Fruit, Vegetable, and Antioxidant Intakes Are Lower in Older Adults with Depression. J Acad Nutr Diet.

[20] Hermsdorff HH, Puchau B, Volp AC, Barbosa KB, Bressan J, Zulet MÁ (2011). Dietary Total Antioxidant Capacity is Inversely Related to Central Adiposity as Well as to Metabolic and Oxidative Stress Markers in Healthy Young Adults. Nutr Metab (Lond).

[21] Puchau B, Zulet MÁ, de Echávarri AG, Hermsdorff HH, Martínez JA (2009). Dietary Total Antioxidant Capacity:A Novel Indicator of Diet Quality in Healthy Young Adults. J Am Coll Nutr.

[22] Serafini M, Del Rio D (2004). Understanding the Association between Dietary Antioxidants, Redox Status and Disease:Is the Total Antioxidant Capacity the Right Tool?. Redox Rep.

[23] Carlsen MH, Halvorsen BL, Holte K, Bøhn SK, Dragland S, Sampson L (2010). The Total Antioxidant Content of More than 3100 Foods Beverages, Spices Herbs and Supplements used Worldwide. Nutr J.

[24] Nascimento-Souza MA, Paiva PG, Martino HS, Ribeiro AQ (2018). Dietary Total Antioxidant Capacity as a Tool in Health Outcomes in Middle-Aged and Older Adults:A Systematic Review. Crit Rev Food Sci Nutr.

[25] Abshirini M, Siassi F, Koohdani F, Qorbani M, Mozaffari H, Aslani Z (2019). Dietary Total Antioxidant Capacity is Inversely Associated with Depression, Anxiety and Some Oxidative Stress Biomarkers in Postmenopausal Women:A Cross-Sectional Study. Ann Gen Psychiatry.

[26] Daneshzad E, Keshavarz SA, Qorbani M, Larijani B, Azadbakht L (2020). Dietary Total Antioxidant Capacity and its Association with Sleep, Stress, Anxiety, and Depression Score:A Cross-Sectional Study among Diabetic Women. Clin Nutr ESPEN.

[27] Miki T, Eguchi M, Kochi T, Akter S, Hu H, Kashino I (2020). Prospective Study on the Association between Dietary Non-Enzymatic Antioxidant Capacity and Depressive Symptoms. Clin Nutr ESPEN.

[28] Milajerdi A, Keshteli AH, Afshar H, Esmaillzadeh A, Adibi P (2019). Dietary Total Antioxidant Capacity in Relation to Depression and Anxiety in Iranian Adults. Nutrition.

[29] de Oliveira NG, Teixeira IT, Theodoro H, Branco CS (2019). Dietary Total Antioxidant Capacity as a Preventive Factor Against Depression in Climacteric Women. Dement Neuropsychol.

[30] Prohan M, Amani R, Nematpour S, Jomehzadeh N, Haghighizadeh MH (2014). Total Antioxidant Capacity of Diet and Serum, Dietary Antioxidant Vitamins Intake, and Serum hs-CRP Levels in Relation to Depression Scales in University Male Students. Redox Rep.

[31] Abshirini M, Siassi F, Koohdani F, Qorbani M, Khosravi S, Hedayati M (2018). Dietary total Antioxidant Capacity is Inversely Related to Menopausal Symptoms:A Cross-Sectional Study among Iranian Postmenopausal Women. Nutrition.

[32] Stroup DF, Berlin JA, Morton SC, Olkin I, Williamson GD, Rennie D (2000). Meta-Analysis of Observational Studies in Epidemiology:A Proposal for Reporting. JAMA.

[33] Page MJ, McKenzie JE, Bossuyt PM, Boutron I, Hoffmann TC, Mulrow CD (2021). The PRISMA 2020 Statement:An Updated Guideline for Reporting Systematic Reviews. BMJ.

[34] NHLBI, NIH (2014). Study Quality Assessment Tools.

[35] Balmus IM, Ciobica A, Antioch I, Dobrin R, Timofte D (2016). Oxidative Stress Implications in the Affective Disorders:Main Biomarkers, Animal Models Relevance, Genetic Perspectives, and Antioxidant Approaches. Oxid Med Cell Longev.

[36] Visentin AP, Colombo R, Scotton E, Fracasso DS, da Rosa AR, Branco CS (2020). Targeting Inflammatory-Mitochondrial Response in Major Depression:Current Evidence and Further Challenges. Oxid Med Cell Longev.

[37] Lehtinen M, Bonni A (2006). Modeling Oxidative Stress in the Central Nervous System. Curr Mol Med.

[38] Villafuerte G, Miguel-Puga A, Rodríguez EM, Machado S, Manjarrez E, Arias-Carrión O (2015). Sleep Deprivation and Oxidative Stress in Animal Models:A Systematic Review. Oxid Med Cell Longev.

[39] Bouayed J (2010). Polyphenols:A Potential New Strategy for the Prevention and Treatment of Anxiety and Depression. Curr Nutr Food Sci.

[40] Milaneschi Y, Bandinelli S, Penninx BW, Corsi AM, Lauretani F, Vazzana R (2012). The Relationship between Plasma Carotenoids and Depressive Symptoms in Older Persons. World J Biol Psychiatry.

[41] Bawaked RA, Schröder H, Ribas-Barba L, Izquierdo-Pulido M, Pérez-Rodrigo C, Fíto M (2017). Association of Diet Quality with Dietary Inflammatory Potential in Youth. Food Nutr Res.

[42] Brighenti F, Valtuena S, Pellegrini N, Ardigo D, Del Rio D, Salvatore S (2005). Total antioxidant Capacity of the Diet is Inversely and Independently Related to Plasma Concentration of High-Sensitivity C-Reactive Protein in Adult Italian Subjects. Br J Nutr.

[43] Valtueña S, Pellegrini N, Franzini L, Bianchi MA, Ardigo D, Del Rio D (2008). Food Selection Based on Total Antioxidant Capacity Can Modify Antioxidant Intake, Systemic Inflammation, and Liver Function without Altering Markers of Oxidative Stress. Am J Clin Nutr.

[44] Saghafian F, Malmir H, Saneei P, Milajerdi A, Larijani B, Esmaillzadeh A (2018). Fruit and Vegetable Consumption and Risk of Depression:Accumulative Evidence from an Updated Systematic Review and Meta-Analysis of Epidemiological Studies. Br J Nutr.

[45] Jacka FN, Kremer PJ, Berk M, de Silva-Sanigorski AM, Moodie M, Leslie ER (2011). A Prospective Study of Diet Quality and Mental Health in Adolescents. PLoS One.

[46] Pereira GA, Domingos AL, de Aguiar AS (2020). Relationship between Food Consumption and Improvements in Circulating Melatonin in Humans:An Integrative Review. Crit Rev Food Sci Nutr.

[47] Domingos AL, Hermsdorff HH, Bressan J (2019). Melatonin Intake and Potential Chronobiological Effects on Human Health. Crit Rev Food Sci Nutr.

[48] Garrido M, Paredes SD, Cubero J, Lozano M, Toribio-Delgado AF, Muñoz JL (2010). Jerte Valley Cherry-Enriched Diets Improve Nocturnal Rest and Increase 6-Sulfatoxymelatonin and Total Antioxidant Capacity in the Urine of Middle-Aged and Elderly Humans. J Gerontol A.

[49] Garrido M, Gonzalez-Gomez D, Lozano M, Barriga C, Paredes SD, Moratinos AB (2013). A Jerte Valley Cherry Product Provides Beneficial Effects on Sleep Quality. Influence on Aging. J Nutr Health Aging.

[50] Howatson G, Bell PG, Tallent J, Middleton B, McHugh MP, Ellis J (2011). Effect of Tart Cherry Juice (*Prunus cerasus*) on Melatonin Levels and Enhanced Sleep Quality. Eur J Nutr.

[51] Parohan M, Anjom-Shoae J, Nasiri M, Khodadost M, Khatibi SR, Sadeghi O (2019). Dietary Total Antioxidant Capacity and Mortality from All Causes, Cardiovascular disease and Cancer:A Systematic Review and Dose-Response Meta-Analysis of Prospective Cohort Studies. Eur J Nutr.

[52] Nayak B, Liu RH, Tang J (2015). Effect of Processing on Phenolic Antioxidants of Fruits, Vegetables, and Grains-a Review. Crit Rev Food Sci Nutr.

[53] Kravitz HM, Kazlauskaite R, Joffe H (2018). Sleep, Health, and Metabolism in Midlife Women and Menopause:Food for Thought. Obstet Gynecol Clin North Am.

[54] Jehan S, Jean-Louis G, Zizi F, Auguste E, Pandi-Perumal SR, Gupta R (2015). Sleep, Melatonin, and the Menopausal Transition:What are the Links?. Sleep Sci.

[55] Lin J, Jiang Y, Wang G, Meng M, Zhu Q, Mei H (2020). Associations of Short Sleep Duration with Appetite-Regulating Hormones and Adipokines:A Systematic Review and Meta-Analysis. Obes Rev.

[56] Singh M (2014). Mood, Food and Obesity. Front Psychol.

[57] Fisberg RM, Marchioni DM, Colucci AC (2009). Assessment of Food Consumption and Nutrient Intake in Clinical Practice. Arq Bras Endocrinol Metabol.

[58] Lettieri-Barbato D, Tomei F, Sancini A, Morabito G, Serafini M (2013). Effect of Plant Foods and Beverages on Plasma Non-Enzymatic Antioxidant Capacity in Human Subjects:A Meta-Analysis. Br J Nutr.

